# Artemisinin derivatives inactivate cancer-associated fibroblasts through suppressing TGF-β signaling in breast cancer

**DOI:** 10.1186/s13046-018-0960-7

**Published:** 2018-11-26

**Authors:** Yuyuan Yao, Qinglong Guo, Yue Cao, Yangmin Qiu, Renxiang Tan, Zhou Yu, Yuxin Zhou, Na Lu

**Affiliations:** 1 State Key Laboratory of Natural Medicines, Jiangsu Key Laboratory of Carcinogenesis and Intervention, School of Basic Medicine and Clinical Pharmacy, China Pharmaceutical University, 24 Tongjiaxiang, Nanjing, 210009 People’s Republic of China; 2 State Key Laboratory Cultivation Base for TCM Quality and Efficacy, Nanjing University of Chinese Medicine, 138 Xinlin Road, Nanjing, 210023 People’s Republic of China

**Keywords:** CAFs, TGF-β1, ART, ARM, ARS, DHA

## Abstract

**Background:**

Cancer-associated fibroblasts (CAFs) are activated fibroblasts associated with cancer. They have an important role in tumor growth and metastasis. Artemisinin (ART) is a sesquiterpene lactone extracted from Chinese herb *qinghao*, and artemether (ARM), artesunate (ARS) and dihydroartemisinin (DHA) were synthesized derivatives of artemisinin, which also have anti-malarial and anti-cancer effects such as artemisinin.

**Methods:**

In this study, we investigated the in-vitro and in-vivo effects of artemisinin derivatives on inactivating cancer-associated fibroblasts and uncovered its underlying mechanism.

**Results:**

We demonstrated that ARS and DHA could revert L-929-CAFs and CAFs from activated to inactivated state in vitro. Mechanically, ARS and DHA could suppress TGF-β signaling to inhibit activation of L-929-CAFs and CAFs, and decreased interaction between tumor and tumor microenvironment. The results showed that ARS and DHA could suppress CAFs-induced breast cancer growth and metastasis in the orthotopic model. Conformably, ARS and DHA suppressed TGF-β signaling to inactivate cancer-associated fibroblasts and inhibit cancer metastasis in vivo*.*

**Conclusions:**

Artemisinin derivatives are potential therapeutic agents for the treatment of breast cancer.

## Background

Breast cancer is a frequent cancer in women and remains a major cause of cancer-associated death in western countries because of lacking effective therapies [1]. Tumorigenesis is a complex process and consists of activating invasion and metastasis, inducing angiogenesis, avoiding immune destruction, and so on [2, 3]. These features required cooperation among various cellular and non-cellular elements of tumors which are known as tumor microenvironment (TME). The cancer-associated fibroblasts (CAFs), immune and inflammatory cells, endothelial cells, bone marrow derived cells, capillaries, basement membrane and extracellular matrix (ECM) surrounding the cancer cells constitute the tumor microenvironment [3, 4]. The study of fibroblasts has grown significantly since late nineteenth century. Fibroblasts associated with cancer are activated and have been termed as cancer-associated fibroblasts (CAFs). Activated fibroblasts may gain further secretory phenotypes, specialized ECM remodeling ability, autocrine activation and dynamic immunomodulatory signaling functions. This process is associated with continuous injurious stimuli, such as the development of cancer lesions [5, 6].

Transforming growth factor-β (TGF-β), platelet-derived growth factor (PDGF), tumor necrosis factor (TNF), stromal cell-derived factor 1 (SDF1) and fibroblast growth factor 2 (FGF2) are key mediators of fibroblast activation in cancer development. TGF-β can recruit and activate fibroblasts in many cancers. Proliferation and invasion of local cancer cells and CAFs are stimulated by TGF-β in tumor microenvironment [5, 6]. The TGF-β pathway is a paradigm of membrane to nucleus signaling by receptor-mediated activation of transcription factors [7, 8]. Transforming growth factor-β1 (TGF-β1) is a member of pleiotropic ligand family which depends on serine/threonine kinase receptors to form heteromeric complexes composed of two ligand binding receptors (TGF-βRII) and two recruited receptors (TGF-βRI). Upon ligand engagement, TGF-βRII transphosphorylates and activates the kinase activity of TGF-βRI resulting in phosphorylation and mobilization of the transcriptional effectors against SMAD proteins [9, 10]. Acting through the SMAD-2/3 axis, TGF-β1 drives the expression of key ECM genes, which are crucial components of developing fibrotic tissue, including collagens, fibronectin and PAI-1. TGF-β1 becomes concentrated in the accumulating ECM, thereby exacerbating the fibrotic response [11].

Artemisinin (ART) is a chemical isolated from the Sweet wormwood (*qinhao*, *Artemisia annua* L., Asteraceae) in 1972 by Youyou Tu at the Chinese Academy of Traditional Chinese Medicine [12, 13]. The unique chemical structure of artemisinin is a sesquiterpene lactone with potent anti-malarial activity. In recent years, by inserting new groups to the parent structure of artemisinin, a series of artemisinin derivatives were synthesized and proved to possess better bioactivity or solubility, including artemether (ARM), artesunate (ARS) and dihydroartemisinin (DHA). Artemisinin and its derivatives are currently considered as the most effective drug in treating cerebral malaria and chloroquine-resistant falciparum malaria [14, 15]. Studies over the past two decades have provided important information on the anti-cancer activity of artemisinin and its derivatives, indicating that this class of compounds may also be effective cancer therapeutic drugs [16, 17].

However, the mechanism of ART, ARM, ARS and DHA in inhibiting cancer metastasis remained unclear, and whether inactivation of CAFs reduced cancer progression and metastasis are still unknown. In the present study, we evaluated the effect of artemisinin and its derivatives on inactivation of cancer-associated fibroblasts. The results showed that ARS and DHA reverted CAFs from activated to inactivated state through suppressing TGF-β pathway, and inhibited cancer growth and metastasis. It suggested that the inactivation of CAFs by artemisinin and its derivatives leads to a decreased interaction between tumor and its ECM, and maybe a promising therapeutic strategy for the treatment of breast cancer.

## Materials and methods

### Reagents

Dimethylsulfoxide (DMSO) was purchased from Sigma-Aldrich (Merck, Darmstadt, Germany). Artemisinin (ART, 98% purity, Aladdin, Shanghai, China), artemether (ARM, 98% purity, Aladdin, Shanghai, China), artesunate (ARS, 98% purity, Aladdin, Shanghai, China), and dihydroartemisinin (DHA, > 98% purity, Aladdin, Shanghai, China) were dissolved in DMSO as a stock solution (0.1 M), stored at − 20 °C, and diluted with DMEM medium (Gibco, Grand Island, NY) before each experiment in in vitro study. Sodium carboxyl methyl cellulose (CMC-Na) was purchased from Sinopharm Chemical Reagent Co., Ltd. (Shanghai, China). ART, ARM, ARS and DHA were prepared as intragastric administration (0.5% CMC-Na) in in vivo study.

CCK-8 cell counting kit and TUNEL FITC apoptosis detection kit were purchased from Vazyme (Vazyme, Nanjing, China). ACCUMAX solution was purchased from Sigma-Aldrich (Merck, Darmstadt, Germany). Mouse TGF-beta 1 ELISA kit was obtained from ABclonal (ABclonal, Wuhan, China). Picrosirius red stain kit was obtained from Yeasen (Yeasen, Shanghai, China). Primary antibody against Ki67 was from Cell Signaling Technology (CST, MA, USA), antibodies against MMP-9, MMP-2, MMP-14, FAP, fibronectin, vimentin, α-SMA, S100A4, p-Smad3 (Ser423/425), Smad3, TGF-β1, and GAPDH were from ABclonal (ABclonal, Wuhan, China). HRP Goat Anti-Mouse IgG (H + L) and HRP Goat Anti-Rabbit IgG (H + L) were from ABclonal (ABclonal, Wuhan, China). Goat anti-Rabbit IgG (H + L) Cross-Adsorbed Secondary Antibody and ProLong™ Gold Antifade Mountant with DAPI were purchased from Thermo (Thermo, Waltham, USA). High-sig ECL Western Blotting Substrate was from Tanon (Tanon, Shanghai, China). TGF-β1 neutralizing antibody was from Abcam plc. (Abcam, Cambridge, UK).

### Animals

Six-week-old female BALB/c nude mice were supplied by Beijing Vital River Laboratory Animal Technology Co., Ltd. (Beijing, China). MMTV-PyMT mice were supplied by Nanjing Biomedical Research Institute of Nanjing University (Nanjing, China). All animals were maintained in a pathogen-free environment (23 ± 2 °C, 55 ± 5% humidity) on a 12 h light/12 h dark cycle with food and water supplied ad libitum throughout the experimental period. Animal study and euthanasia was carried out in strict accordance with the recommendations in the Guide for the Care and Use of Laboratory Animals of the National Institutes of Health. The protocol was approved by the Committee on the Ethics of Animal Experiments of the China Pharmaceutical University.

### Cell culture

Mouse breast carcinoma 4 T1 cells and mouse fibroblast L-929 cells were kindly provided by Cell Bank, Chinese Academy of Sciences. Cells were cultured in DMEM medium (Gibco, Grand Island, NY) containing 10% fetal bovine serum (Gibco), 100 U/ml penicillin, and 100 μg/ml streptomycin, in a humidified atmosphere containing 5% CO_2_ at 37 °C.

Conditioned media (CM) was collected from the supernatant of 4 T1 cells labeled with luciferase, and CM was used for conditioned culture with L-929 cells in different experiments. L-929 cells were cultured in CM for 48 h to be activated and possessed with main characteristics of cancer-associated fibroblasts, then set up the conditioned culture model of L-929-CAFs.

### Cancer-associated fibroblasts isolation [18, 19]

Cancer-associated fibroblasts (CAFs) were isolated from breast tumors from MMTV-PyMT mice. Fresh tumor tissues were dissected in a sterile environment, and washed several times with PBS (4 °C pre-cooled, containing 200 U/ml penicillin and 200 μg/ml streptomycin). The tissue membrane and necrosis were removed with sterile ophthalmic scissors and forceps. The remaining tissues were minced into smaller pieces and treated 4 h with ACCUMAX in a sterile environment at 37 °C. Single cell suspensions were prepared and cultured in DMEM medium containing 10% fetal bovine serum, 100 U/ml penicillin, and 100 μg/ml streptomycin, in a stable environment containing 5% CO_2_ at 37 °C. After 24 h, the non-adherent cells were washed away and the adherent cells were cultured in fresh medium. The medium was changed every 2 to 4 days until the cells proliferated apparently.

### Cell viability assay

CCK-8 cell counting assay was carried out to measure the effect of ART, ARM, ARS or DHA on cell viability. Cells were plated at a density of 5 × 10^3^ cells per well in 96-well plates. After 24 h culture, the cells were exposed to ART, ARM, ARS or DHA for 24 h in a 5% CO_2_ incubator at 37 °C. L-929-CAFs were treated with 50 μM of ART, ARM, ARS or DHA, CAFs were treated with 30 μM of ART, ARM, ARS or DHA. Then, CCK-8 solution was added to the medium and incubated at 37 °C for additional 4 h. The absorbance was measured at 450 nm.

### Western blot

Cells were harvested after pretreatment of ART, ARM, ARS or DHA for 24 h. Western blot was performed as previously described [20]. The membrane was blocked with 3% no fat milk in PBST at 37 °C for 1 h and incubated with the indicated antibodies overnight at 4 °C, and then with HRP Goat Anti-Mouse IgG (H + L) or HRP Goat Anti-Rabbit IgG (H + L) secondary antibody for 1 h at 37 °C. The samples were visualized with High-sig ECL Western Blotting Substrate (Tanon, Shanghai, China) and Fully Automatic Chemiluminescence Image Analysis System (Tanon, Shanghai, China).

### Immunofluorescence

Cells were exposed to ART, ARM, ARS or DHA for 24 h in a 5% CO_2_ incubator at 37 °C before being washed with PBS twice. L-929-CAFs were treated with 50 μM of ART, ARM, ARS or DHA, CAFs were treated with 30 μM of ART, ARM, ARS or DHA. Cells were fixed with 4% paraformaldehyde for 30 min, permeabilized for 15 min in 0.2% Triton X-100 on ice, and blocked with 3% BSA for 2 h. Then the cells were incubated with the indicated antibodies overnight at 4 °C. After washed with PBS for three times, cells were exposed to Goat anti-Rabbit IgG (H + L) Cross-Adsorbed Secondary Antibodies (Thermo, Waltham, USA) for 1 h. Then cells were washed with PBS for three times and stained with ProLong™ Gold Antifade Mountant with DAPI. Finally, the slides were photographed with a confocal laser scanning microscope (Fluoview FV1000, Olympus, Tokyo, Japan).

### Cytokines detection by ELISA

The concentration of cytokines in supernatant was detected by Mouse TGF-beta 1 ELISA kit (ABclonal, Wuhan, China) according to the manufacturer’s recommendations [21, 22]. The absorbance was measured at 450 nm.

### Cell directional migration assay

Transwell chambers (12 mm in diameter, 8 μm pore-size, Millipore, Billerica, MA) were used to evaluate the chemotactic motility of 4 T1 cells in cancer environment [22]. L-929-CAFs or CAFs were incubated in 24-well plates, 600 μl medium containing ART, ARM, ARS or DHA was added to the lower compartment. L-929-CAFs were treated with 50 μM of ART, ARM, ARS or DHA, CAFs were treated with 30 μM of ART, ARM, ARS or DHA. 4 T1 cells labeled with luciferase were collected in serum-free medium at a final concentration of 3 × 10^5^ cells/ml. 400 μl cell suspensions were then placed in the upper transwell chamber. After incubation for 24 h, cells on the upper surface were removed, and directional migration cells on the lower surface were fixed with 100% methanol and stained with hematoxylin and eosin. Then quantified by manual counting and three randomly chosen fields were analyzed for each group.

### Cell invasion assay

Cell invasion assay was performed as similar with cell directional migration assay above except that the matrigel (Corning, NY, USA) diluted by serum-free medium (1:8) was coated on the upper transwell chambers at 37 °C for 1 h in advance [21]. After 24 h incubation, the invasion cells were fixed, stained and counted by microscope.

### Animal model

Orthotopic injections were performed following the previous study with minor modifications [21, 23, 24]. 1 × 10^4^/25 μl 4 T1 cells labeled with luciferase, or 1 × 10^4^/25 μl 4 T1-luciferase cells plus 3 × 10^4^/25 μl L-929 cells were suspended in 25 μl matrigel on ice, and then the cell suspension was quickly injected into the fourth Mammary Fat Pad (MFP). Animal was observed for 30 min until fully recovery. After 28 days, the mice injected 4 T1 cells were assigned to the negative control group (8 mice/group, gavage of 0.5% CMC-Na at a frequency of once every day), the mice injected 4 T1-luciferase cells plus L-929 cells were randomly divided into six groups (8 mice/group): the control group (gavage of 0.5% CMC-Na at a frequency of once every day), the paclitaxel-positive group (intraperitoneal injection of 10 mg/kg paclitaxel at a frequency of one time every 2 day), the ART-treated group (gavage of 100 mg/kg ART at a frequency of once every day); the ARM-treated group (gavage of 100 mg/kg ARM at a frequency of once every day); the ARS-treated group (gavage of 100 mg/kg ARS at a frequency of once every day); the DHA-treated group (gavage of 100 mg/kg DHA at a frequency of once every day). The weight, tumor width (W) and Length (L) were measured by an average of three days. The tumor volume (V) was estimated with the formula: V = 1/2 × L × W^2^. 28 days later, mice bearing tumors were conducted optical imaging to determine the luciferase expression in the mouse model using the IVIS Lumina Bioluminescence Imaging System, then the nude mice were killed and the tumor xenografts were segregated and measured. Visceral tissue resected from mice were fixed in formalin and tested with H&E staining.

This study was approved in SPF Animal Laboratory of China Pharmaceutical University. In all experiments, the ethics guidelines for investigations in conscious animals were followed, with approval from the local Ethics Committee for Animal Research.

### Immunohistochemistry

The expression of Ki67, α-SMA, vimentin and TGF-β1 of tumor tissues in nude mice model were assessed to the manufacturer’s instructions [25], using a SPlink detection kit (Biotin-Streptavidin HRP Detection Systems). All reagents used in the experiments were supplied by ZSGB-Bio Co., Beijing, China. Observation and picture were taken under a light microscope.

### TUNEL assay

Apoptosis induction in the tissue specimen was analyzed by TUNEL test. It was performed as per instructions given in TUNEL FITC apoptosis detection kit (Vazyme, Nanjing, China). The slides were photographed with a confocal laser scanning microscope (Fluoview FV1000, Olympus, Tokyo, Japan).

### Sirius red staining assay

Tissue sections were stained using picrosirius red stain kit (Yeasen, Shanghai, China) according to the manufacturer’s instructions. Observation and picture were taken under a light microscope.

### Statistical analysis

The data were obtained from at least three independent experiments and all data in different experimental groups were expressed as the mean ± SD. The comparisons were made relative to CM-induced group or control group in vitro*,* 4 T1 + L-929 group in vivo. Differences between groups were tested with One-Way ANOVA analysis of variance and Dunnett’s post hoc test. The changes in tumor volume over time were tested using a random effects mixed model. Metastasis incidence rates were evaluated using percentages of animals with metastases. The significance of differences is indicated at *p < 0.05 and **p < 0.01.

## Results

### ARS and DHA reverted L-929-CAFs and CAFs from activated to inactivated state

**Fig. 1:**
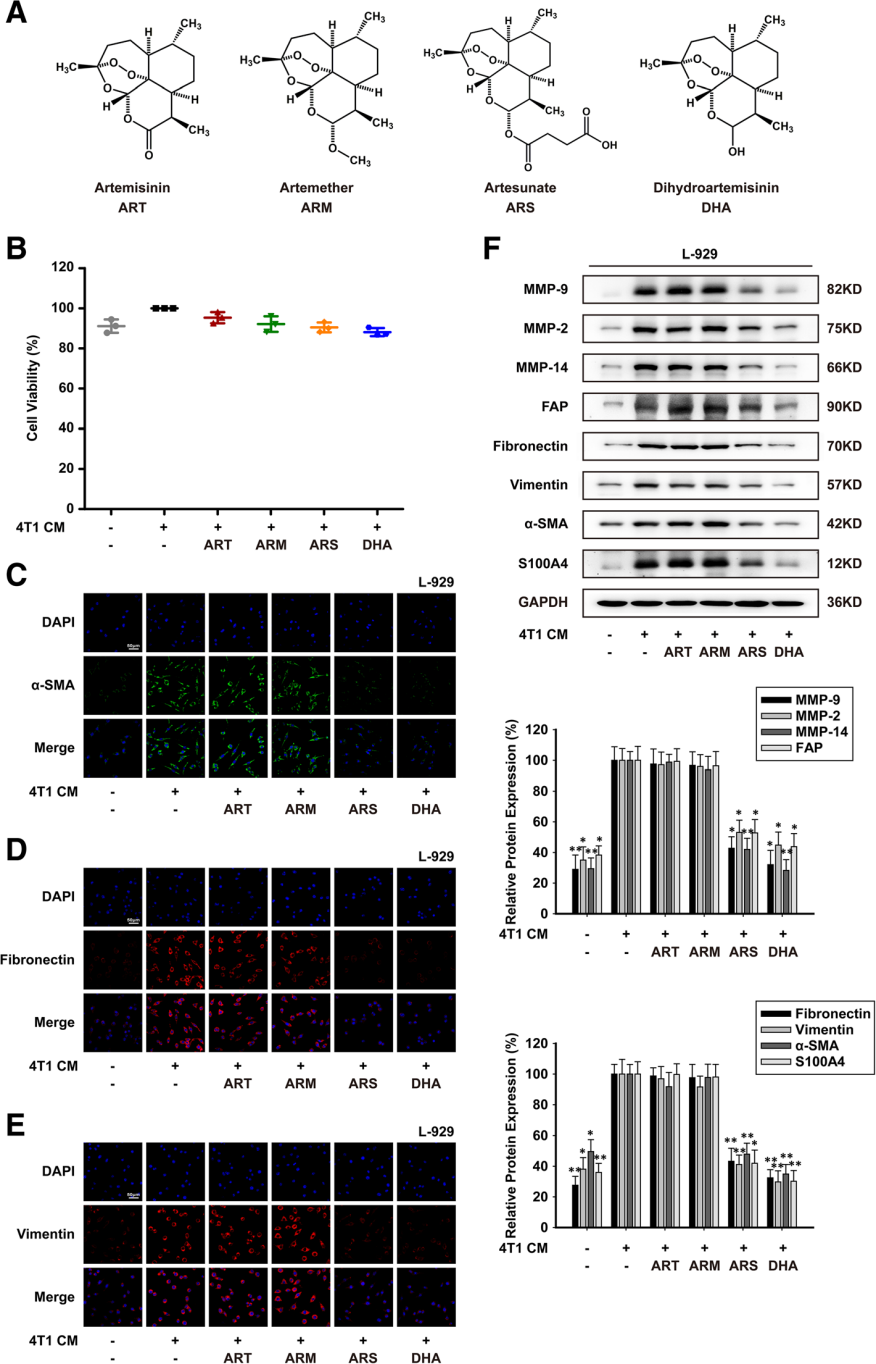
ARS and DHA reverted L-929-CAFs from activated to inactivated state. L-929-CAFs were exposed to 50 μM of ART, ARM, ARS or DHA for 24 h. (**a**) Chemical structure of ART, ARM, ARS and DHA. (**b**) Effect of ART, ARM, ARS and DHA on cell viability by CCK-8 cell counting assay. (**c**-**e**) Immunofluorescence staining assay for α-SMA, fibronectin and vimentin were performed to test the effect of ART, ARM, ARS and DHA on CAFs related biomarker proteins (image magnification: 400×). (**f**) The expression of MMP-9, MMP-2, MMP-14, FAP, fibronectin, vimentin, α-SMA and S100A4 proteins in the cells were analyzed by western blot using specific antibodies. Each experiment was performed at least three times. Data are presented as mean ± SD. *p < 0.05 compared with CM-induced group; **p < 0.01 compared with CM-induced group

**Fig. 2:**
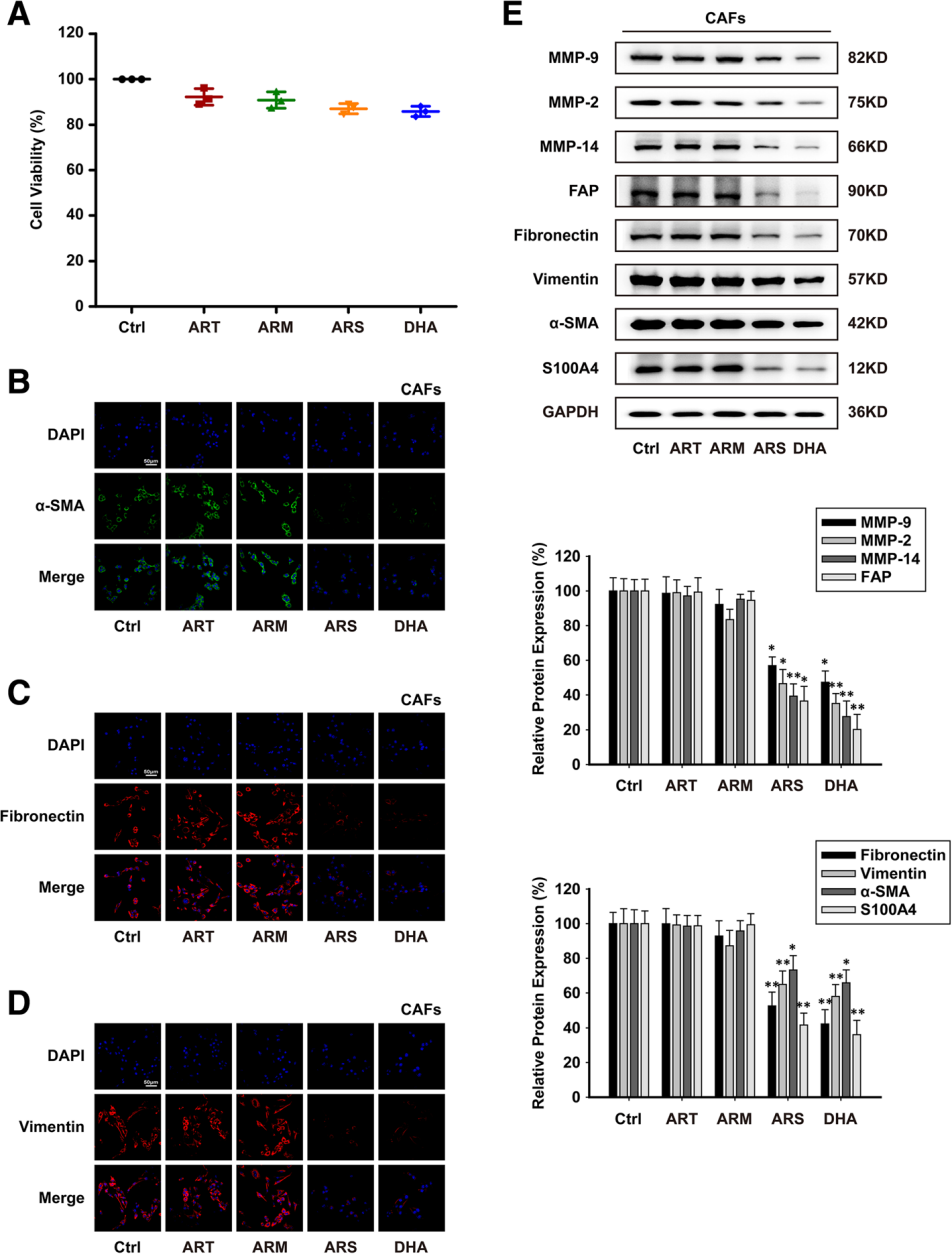
ARS and DHA reverted CAFs from activated to inactivated state. CAFs were exposed to 30 μM of ART, ARM, ARS or DHA for 24 h. (**a**) Effect of ART, ARM, ARS and DHA on cell viability by CCK-8 cell counting assay. (**b**-**d**) Immunofluorescence staining assay for α-SMA, fibronectin and vimentin were performed to test the effect of ART, ARM, ARS and DHA on CAFs related biomarker proteins (image magnification: 400×). (**e**) The expression of MMP-9, MMP-2, MMP-14, FAP, fibronectin, vimentin, α-SMA and S100A4 proteins in the cells were analyzed by western blot using specific antibodies. Each experiment was performed at least three times. Data are presented as mean ± SD. *p < 0.05 compared with the control group; **p < 0.01 compared with the control group

The chemical structures of ART, ARM, ARS and DHA were shown in Fig. 1a. In order to exclude the proliferation-inhibition effect of ART, ARM, ARS and DHA on L-929-CAFs and CAFs, CCK-8 cell counting assay was implemented. As shown in Figs. 1b and 2a, 50 μM or 30 μM of ART, ARM, ARS or DHA had little effect on cell viability of L-929-CAFs and CAFs. Therefore, these concentrations were then applied to all subsequent experiments in vitro.

CAFs were identified by morphological characteristics and specific markers expression [26, 27]. In order to investigate the effects of CM induce-conditioned culture model, immunofluorescence staining assay and western blot assay were used to test the specific markers expression such as MMP-9, MMP-2, MMP-14, FAP, fibronectin, vimentin, α-SMA and S100A4. The results showed that these proteins were upregulated under 4 T1 CM, which suggested that 4 T1 CM could activate L-929 cells to L-929-CAFs in conditioned culture system (Fig. 1c-f). As shown in Figs. 1c-f and 2b-e, treatment of L-929-CAFs and CAFs with ARS or DHA decreased the expression of activation proteins, but ART and ARM had no effect on their expression, indicating that ARS and DHA could inhibit CAFs activation.

### ARS and DHA suppressed TGF-β pathway to inhibit activation of L-929-CAFs and CAFs

**Fig. 3:**
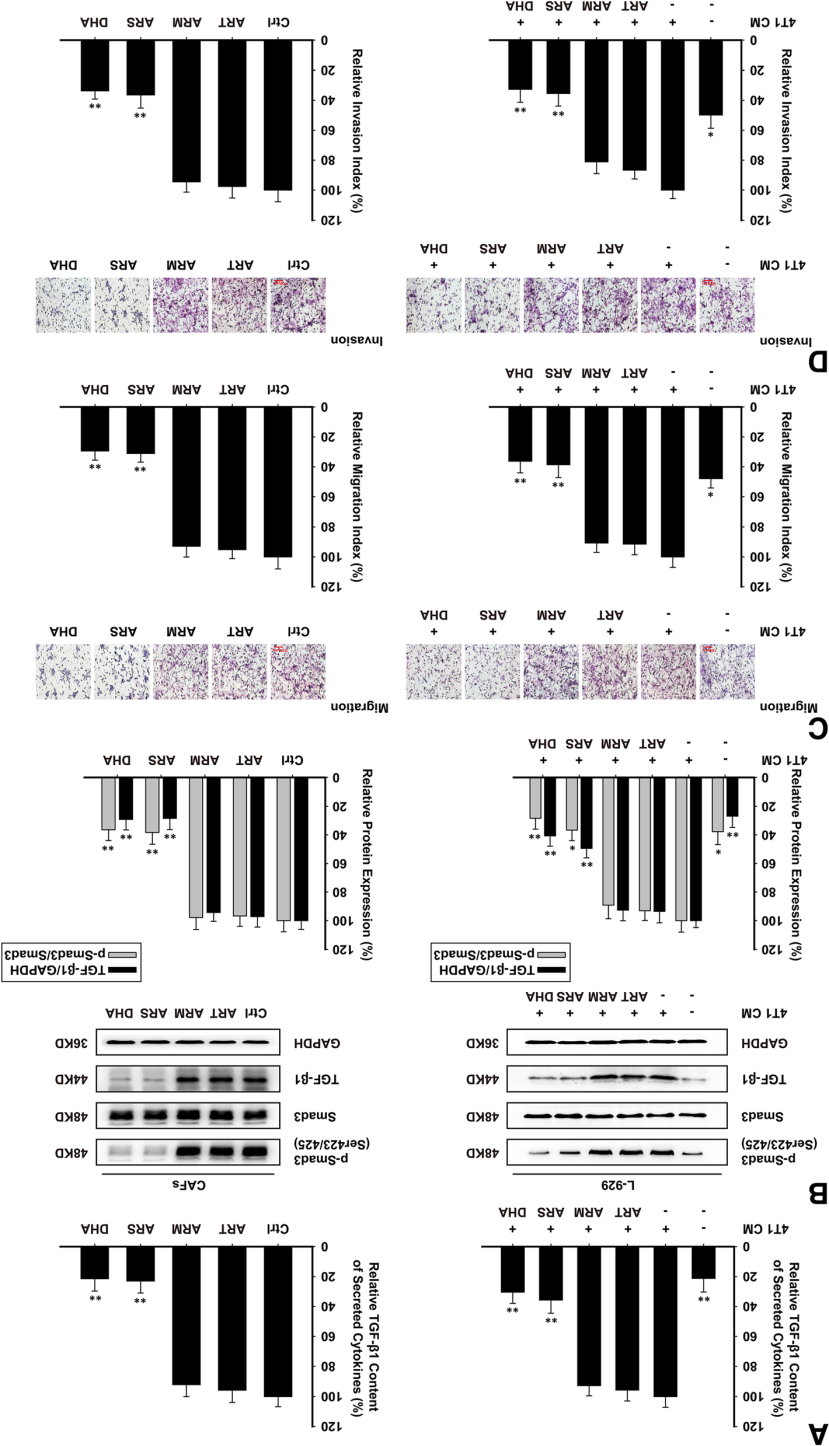
ARS and DHA suppressed TGF-β pathway to inhibit activation of L-929-CAFs and CAFs. L-929-CAFs and CAFs were treated with 50 μM or 30 μM of ART, ARM, ARS or DHA for 24 h respectively. (**a**) The effect of ART, ARM, ARS and DHA on TGF-β1 content of secreted cytokines. (**b**) The expression of p-Smad3 (Ser423/425), Smad3 and TGF-β1 proteins in the cells were analyzed by western blot using specific antibodies. (**c**) The migration ability was evaluated by a cell directional migration assay (image magnification: 200×). (**d**) The invasive ability was evaluated by a matrigel-coated transwell invasion assay (image magnification: 200×). Each experiment was performed at least three times. Data are presented as mean ± SD. *p < 0.05 compared with CM-induced or control group; **p < 0.01 compared with CM-induced or control group

By Elisa assay and western blot analysis, we found that the secretion level of TGF-β1 in L-929-CAFs was increased compared to normal L-929 cells, and ARS and DHA could reduce the protein expression of p-Smad3 (Ser423/425) and TGF-β1 both in L-929-CAFs and CAFs, but no significant differences were found in ART and ARM groups (Fig. 3a and b). Together these results provided important insight that ARS and DHA may inactive CAFs through TGF-β1.

Prior studies have noted the importance of activated fibroblasts, which can promote tumor development and invasion via their active secretome, including growth factors and extracellular matrix (ECM) [6, 28]. Cell directional migration assay and cell invasion assay were used to evaluate the effects of artemisinin and its derivatives on 4 T1 cells migration and invasion in the presence of CAFs. As shown in Fig. 3c and d, L-929-CAFs could increase 4 T1 cells migration and invasion in co-culture system. When L-929-CAFs and CAFs were treated with ARS and DHA, the migration and invasion of 4 T1 cells were decreased dramatically compared with untreated CAFs. However, ART and ARM could not inhibit the migration and invasion of 4 T1 cells. These results demonstrated that the promoting functions of CAFs on migration and invasion were impaired by ARS and DHA.

### ARS and DHA could not further inactivate L-929-CAFs and CAFs with TGF-β1 neutralizing antibody

**Fig. 4:**
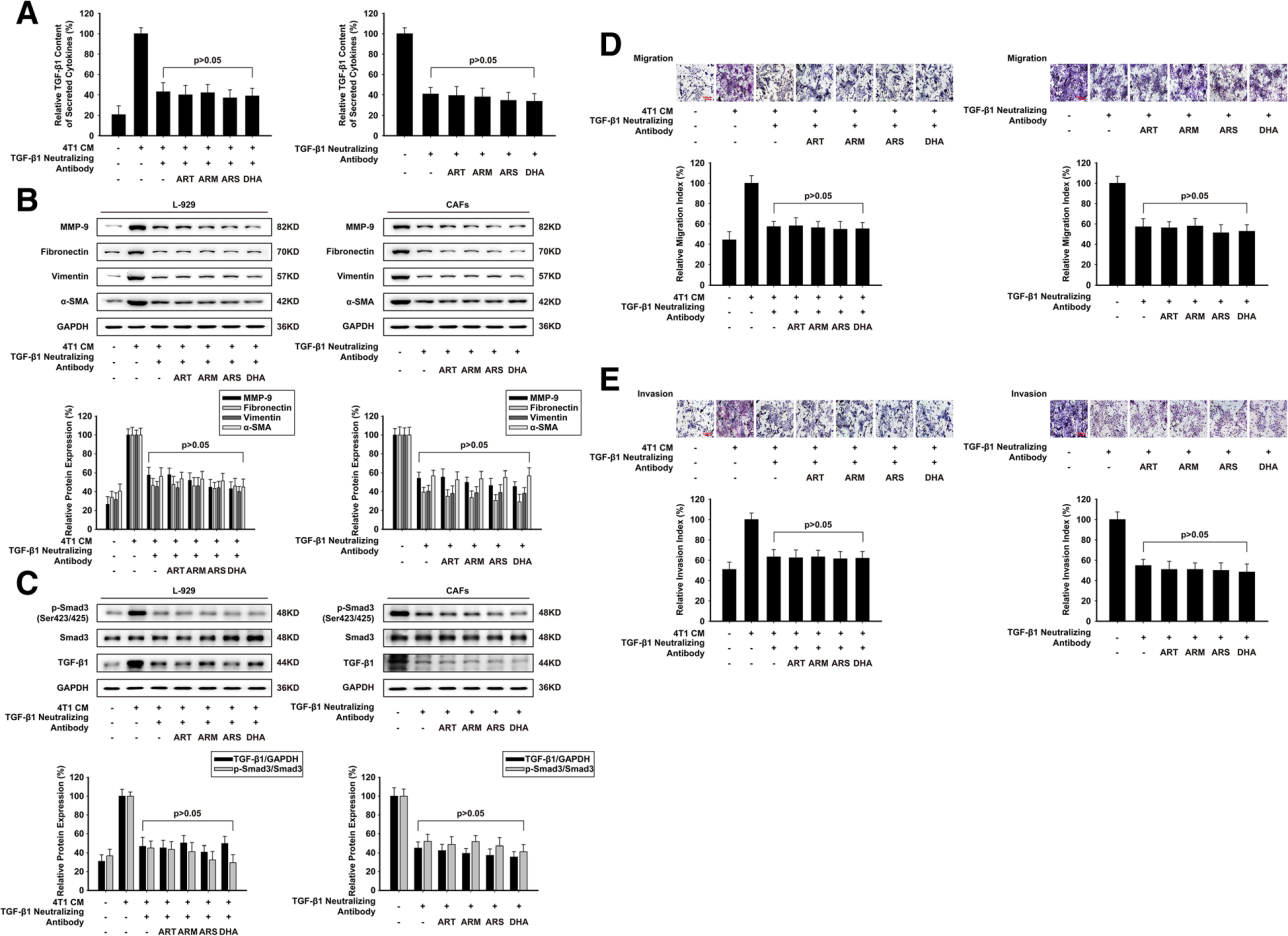
ARS and DHA could not further inactivate L-929-CAFs and CAFs with TGF-β1 neutralizing antibody. L-929-CAFs and CAFs were treated with 50 μM or 30 μM of ART, ARM, ARS or DHA for 24 h respectively. TGF-β1 neutralizing antibody was added to the culture system to exhaust secreted TGF-β1. (**a**) The effect of ART, ARM, ARS and DHA on TGF-β1 content of secreted cytokines. (**b**) The expression of MMP-9, fibronectin, vimentin, and α-SMA proteins in the cells were analyzed by western blot using specific antibodies. (**c**) The expression of p-Smad3 (Ser423/425), Smad3 and TGF-β1 proteins in the cells were analyzed by western blot using specific antibodies. (**d**) The migration ability was evaluated by a cell directional migration assay (image magnification: 200×). (**e**) The invasive ability was evaluated by a matrigel-coated transwell invasion assay (image magnification: 200×). Each experiment was performed at least three times. Data are presented as mean ± SD. *p < 0.05 compared with CM-induced or control group; **p < 0.01 compared with CM-induced or control group

To confirm the significant roles of TGF-β1 in the conditioned culture or co-culture system, we blocked the interaction of TGF-β1 using TGF-β1 neutralizing antibody. As shown in 4A, 4B and 4C, Elisa assay and western blot assay proved that TGF-β1 neutralizing antibody could reduce the expression of TGF-β1, and inhibited the activation effect of CAFs. Artemisinin and its derivatives could not further downregulate specific markers protein expression or suppress TGF-β pathway with TGF-β1 neutralizing antibody. As shown in Fig. 4d and e, the presence of TGF-β1 neutralizing antibody in the co-culture system reduced the number of migrated and invasive 4 T1 cells. Artemisinin and its derivatives could not strength these inhibition effects when TGF-β1 neutralizing antibody was added to the co-culture system.

### ARS and DHA suppressed CAFs-induced cancer growth and metastasis in vivo

**Fig. 5:**
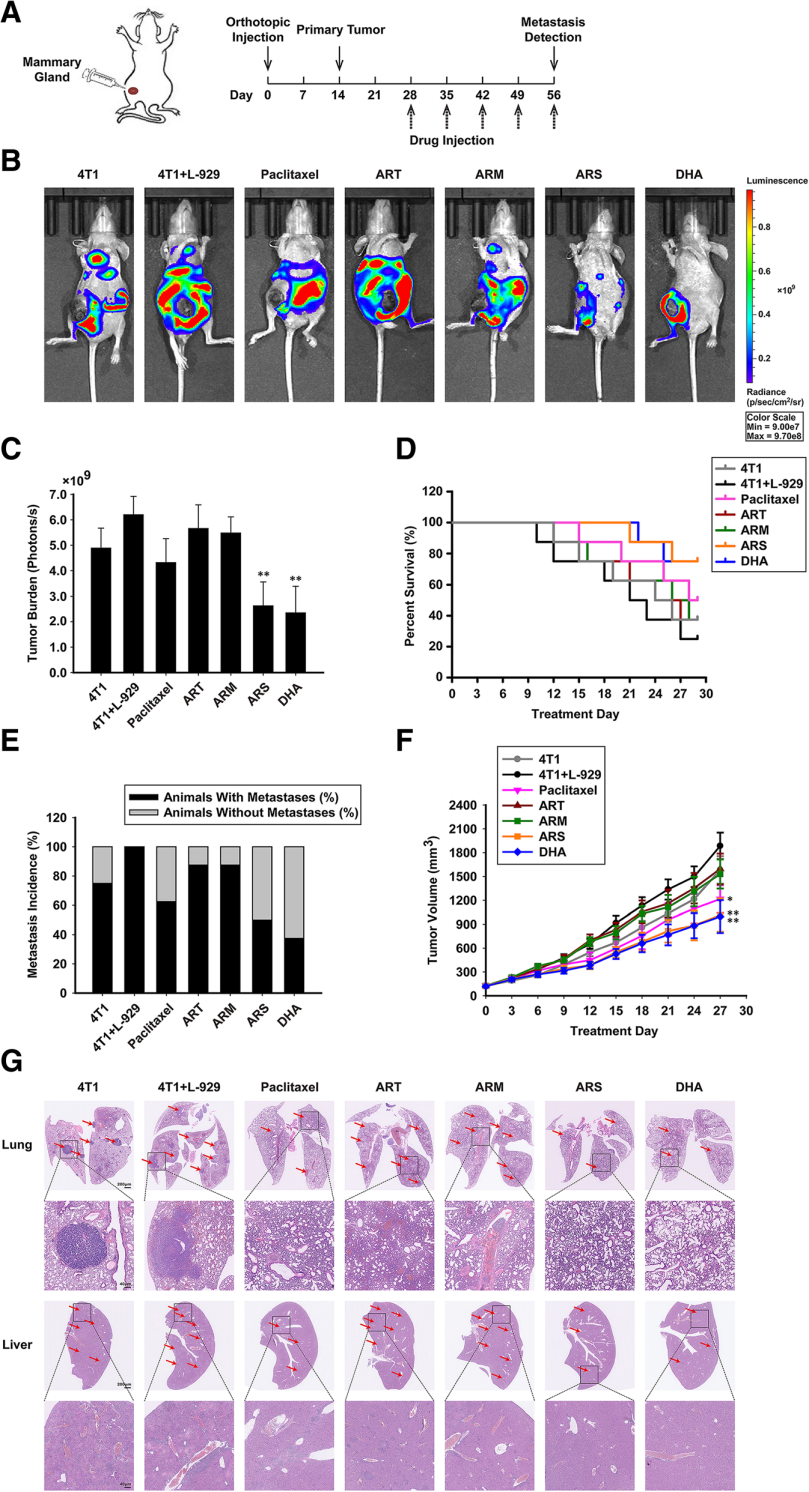
ARS and DHA suppressed CAFs-induced cancer growth and metastasis in vivo. (**a**) Diagram shows the experimental course of orthotopic model (*n* = 8). (**b**-**c**) Representative images and quantification bioluminescence of breast tumor bearing mice treated with paclitaxel (10 mg/kg), ART (100 mg/kg), ARM (100 mg/kg), ARS (100 mg/kg) and DHA (100 mg/kg). (**d**) Kaplan-Meier survival curves for breast tumor bearing mice. (**e**) Probability of metastasis for breast tumor bearing mice. (**f**) Effect of paclitaxel (10 mg/kg), ART (100 mg/kg), ARM (100 mg/kg), ARS (100 mg/kg) and DHA (100 mg/kg) on tumor growth was investigated in the orthotopic model. (**g**) H&E stained lungs and livers of mice to confirm the presence of micrometastases. Each experiment was performed at least three times. Data are presented as mean ± SD. *p < 0.05 compared with 4 T1 + L-929 group; **p < 0.01 compared with 4 T1 + L-929 group

The breast cancer orthotopic model was used to see whether ART, ARM, ARS and DHA could affect CAF-induced tumor burden and metastatic effect in vivo (Fig. 5a). It could be seen from the data in Fig. 5b-g that the presence of CAFs generated larger tumors, more bioluminescence signals and metastatic lesions than that did not have CAFs injected. As shown in Fig. 5b and c, the bioluminescence signals after 28-day treatment were significantly decreased by ARS and DHA. ARS and DHA significantly prolonged life span of tumor bearing mice compared with the other groups (Fig. 5d). As can be seen from Fig. 5e, the probabilities of metastasis induced by CAFs were down-regulated by ARS (50%) and DHA (37.5%), while that of paclitaxel, ART and ARM were 62.5, 87.5 and 87.5%. As shown in Fig. 5f, during 28-day treatment, ARS and DHA reduced tumor volume more apparently than paclitaxel, ART and ARM. In the orthotopic model, cancer cells could metastasize to several organs, including brain, lung, liver and bone. Afterwards, we analyzed the metastasis in lung and liver according to the pathological section. The results showed that ARS and DHA could suppress the formation of metastases in lung and liver (Fig. 5g).

### ARS and DHA inactivated cancer-associated fibroblasts through suppressing TGF-β signaling in vivo

**Fig. 6:**
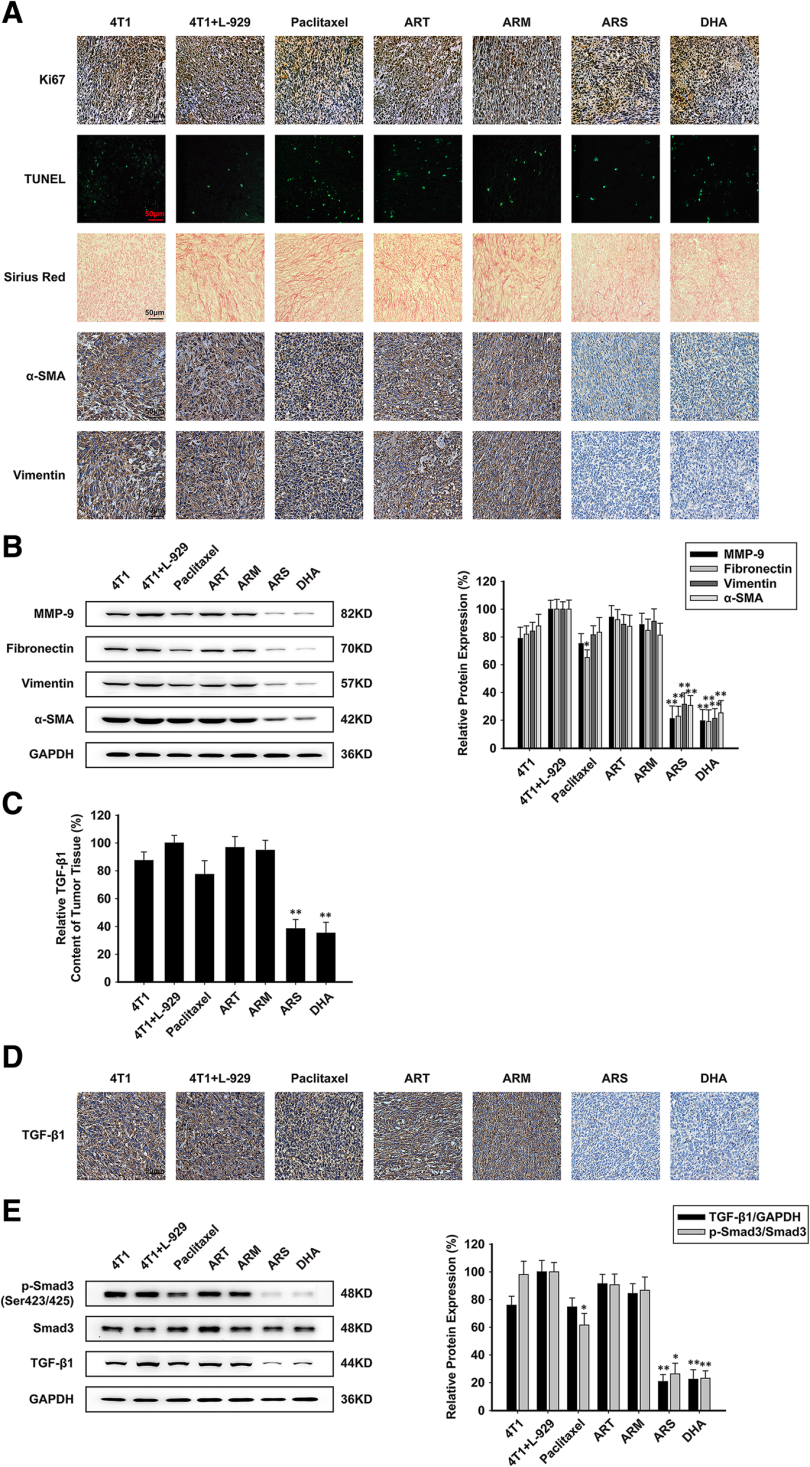
ARS and DHA inactivated CAFs through suppressing TGF-β signaling in vivo. (**a**) TUNEL, sirius red staining and immunohistochemical detection of Ki67, α-SMA and vimentin protein levels on CAFs related biomarker in orthotopic site (image magnification: 400×). (**b**) The expression of MMP-9, fibronectin, vimentin, and α-SMA proteins were analyzed in orthotopic site by western blot using specific antibodies. A GAPDH antibody was used to check equivalent protein loading. (**c**) The effect of paclitaxel, ART, ARM, ARS and DHA on TGF-β1 content of tumor tissue. (**d**) Immunohistochemical detection of TGF-β1 protein levels in orthotopic site (image magnification: 400×). (**e**) The expression of p-Smad3 (Ser423/425), Smad3 and TGF-β1 proteins were analyzed in orthotopic site by western blot using specific antibodies. Each experiment was performed at least three times. Data are presented as mean ± SD. *p < 0.05 compared with 4 T1 + L-929 group; **p < 0.01 compared with 4 T1 + L-929 group

As can be seen from Fig. 6a, we investigated the effect of ART, ARM, ARS and DHA on CAF-induced orthotopic tumors by Ki67 detection and TUNEL assay. The proportions of Ki67 positive cells in tumor tissues were decreased at a small fraction after treatment with paclitaxel, ART, ARM, ARS and DHA. TUNEL assay performed to detect apoptotic cells in tumor tissues showed that ART, ARM, ARS and DHA could not induce DNA damage of tumor tissues. Therefore, we deduced that ART, ARM, ARS and DHA could not induce tumor apoptosis and limited cell proliferation inhibition in vivo. The results of sirius red staining assay, immunohistochemistry and western blot assay showed that ARS and DHA treatment decreased collagen in the tissue, and the expression of specific markers such as MMP-9, fibronectin, vimentin and α-SMA (Fig. 6a and b). By Elisa assay, we found that the secretion level of TGF-β1 in CAF-induced tumors was increased, compared with that did not have CAFs injected (Fig. 6c). Meanwhile, the protein expression of p-Smad3 (Ser423/425) and TGF-β1 were also increased in CAF-induced tumors (Fig. 6d and e). Consistently, as shown in Fig. 6c, d and e, the Elisa assay, immunohistochemistry assay and western blot assay verified that ARS and DHA could reduce the protein expression of p-Smad3 (Ser423/425) and TGF-β1, but ART and ARM had no effect on the protein expression.

## Discussion

Artemisinin (ART) is a sesquiterpene lactone with a 1,2,4-trioxane ring system, extracted from the Chinese herb *qinghao*. Based on the sesquiterpene lactone template, three generations of artemisinin-like semisynthetic and fully synthetic endoperoxide compounds including artemether (ARM), artesunate (ARS) and dihydroartemisinin (DHA) were developed to improve the efficacy and tolerability [29]. Previous studies reported that the effective drug concentrations on cancer cells are usually higher than those to kill malaria parasites. The endoperoxide moiety of artemisinin structure is responsible for its anti-malarial and anti-cancer effects. Reducing heme or ferrous iron can activate the endoperoxide bond and engender the cytotoxic carbon-centered radicals. However, the absence of endoperoxide moiety does not completely abolish the anticancer activity but significantly reduces cytotoxicity, which suggested that the anti-cancer activity of artemisinin and its derivatives may involve other alternative mechanisms [29–32]. In this study, we demonstrated that artemisinin derivatives could inactivate cancer-associated fibroblasts to inhibit cancer growth and metastasis in breast cancer for the first time.

Cancer-associated fibroblasts (CAFs) have an important role in tumor growth and metastasis, such as stimulating angiogenesis, cell proliferation, migration and invasion. CAFs could release growth factors and cytokines, produce metalloproteinases (MMPs) and down-regulate of CD36 [3, 33, 34]. CAFs could affect ECM stiffness at primary tumors, enhancing cancer cell migration and invasion by inducing epithelial-mesenchymal transition (EMT) [3, 35, 36]. Specific markers are used to identify CAFs, such as α-smooth muscle actin (α-SMA), fibroblastspecific protein-1 (FSP1, also called S100A4), fibroblast activating protein (FAP), vimentin, platelet-derived growth factor receptor-α (PDGFR-α), platelet-derived growth factor receptor-β (PDGFR-β), chondroitin sulfate proteoglycan neuron-glial antigen-2 (NG2), and so on [26]. Therefore, immunofluorescence and western blot assay were used to measure the effects of artemisinin and its derivatives on inactivating L-929-CAFs and CAFs in vitro. We found that ARS and DHA could decrease the expression of specific markers to inhibit CAFs activation, which was significant to evaluate the inactivation effects of artemisinin and its derivatives.

TGF-β binds to the type II receptor on the cell surface, recruiting the TGF-β type I receptor (TGF-βRI). Activated TGF-βRI phosphorylates the COOH-terminal regions of Smad2 and Smad3, forming a complex with Smad4 to translocate from cytoplasm to nucleus, regulating gene expression in cell proliferation, differentiation, migration and ECM production. Anti-TGF-β therapies have also been evaluated in cancer treatment because they could interfere with different components of the tumor environment [37–39]. Our study demonstrated that ARS and DHA suppressed TGF-β signaling in CAFs through decreased secretion of TGF-β1. In addition, the increased expression of TGF-β1 by CM induce-conditioned culture system was also decreased by ARS and DHA. When CAFs were treated with TGF-β1 neutralizing antibody, the effects of artemisinin and its derivatives on specific markers protein were not decreased significantly, and the inhibitions of TGF-β signaling were not enhanced, which indicated that artemisinin derivatives inactivated CAFs may depend on TGF-β1 expression.

If fibroblasts expressed activation protein and secretory protein in tumor environment to become CAFs, the CAFs could not return to the quiescent stage [6]. Consequently, developing drugs targeted CAFs at quiescent stage maybe an attractive therapeutic strategy, which can disrupt the tumor-stroma cross-talk and inhibit tumor progression. We found that although CAFs could induce 4 T1 cells migration and invasion, artemisinin derivatives could also decrease the migration and invasion of 4 T1 cells. In addition, artemisinin derivatives could not cause more reductive effects of migration and invasion when TGF-β1 neutralizing antibody was added to the co-culture system. These findings suggested that artemisinin derivatives could decrease the cross-talk between CAFs and cancer cells to inhibit the tumor metastasis.

The breast cancer orthotopic model in BALB/c nude mice was used to see whether artemisinin and its derivatives affected CAF-induced tumor burden and metastatic effect in vivo*.* Considering that oral drug administration is safer and more convenient, we adopted gavage instead of intravenous injection to reduce the toxicity in BALB/c nude mice. We observed that the presence of CAFs could induce larger cancer volume and more metastatic lesions. The results further verified that artemisinin and its derivatives suppressed TGF-β signaling to reduce CAFs-induced cancer growth and metastasis in vivo*.*

The present study was subject to a number of potential methodological weaknesses, such as co-cultures and xenograft mouse models. These experimental models are fundamental to preclinical cancer research, but substantial gaps exist between the results of these experiments and those obtained in clinical settings [27]. Further studies are required in order to validate the effectiveness of artemisinin derivatives in cancer in clinical study.

## Conclusions

In conclusion, ARS and DHA could suppress TGF-β signaling, thus inactivated cancer-associated fibroblasts, and finally inhibited cancer growth and metastasis. Therefore, artemisinin derivatives might be potential therapeutic agents for the treatment of breast cancer.

## Abbreviations

ARM: Artemether
ARS: Artesunate
ART: Artemisinin
CAFs: Cancer-associated fibroblasts
DHA: Dihydroartemisinin
ECM: Extracellular matrix
EMT: Epithelial-mesenchymal transition
FAP: Fibroblast activating protein
FGF2: Fibroblast growth factor 2
FSP1 (S100A4): Fibroblast specific protein-1
MFP: Mammary fat pad
MMPs: Metalloproteinases
NG2: Chondroitin sulfate proteoglycan neuron-glial antigen-2
PDGF: Platelet-derived growth factor
SDF1: Stromal cell-derived factor 1
TGF-β: Transforming growth factor-β
TME: Tumor microenvironment
TNF: Tumor necrosis factor
TV: Tumor volume
α-SMA: α-smooth muscle actin
