# Correction to: Artemisinin derivatives inactivate cancer-associated fibroblasts through suppressing TGF-β signaling in breast cancer

**DOI:** 10.1186/s13046-019-1441-3

**Published:** 2019-11-05

**Authors:** Yuyuan Yao, Qinglong Guo, Yue Cao, Yangmin Qiu, Renxiang Tan, Zhou Yu, Yuxin Zhou, Na Lu

**Affiliations:** 1 State Key Laboratory of Natural Medicines, Jiangsu Key Laboratory of Carcinogenesis and Intervention, School of Basic Medicine and Clinical Pharmacy, China Pharmaceutical University, 24 Tongjiaxiang, Nanjing, 210009 People’s Republic of China; 2 State Key Laboratory Cultivation Base for TCM Quality and Efficacy, Nanjing University of Chinese Medicine, 138 Xinlin Road, Nanjing, 210023 People’s Republic of China

**Correction to: J Exp Clin Cancer Res (2018) 37: 282**

**https://doi.org/10.1186/s13046-018-0960-7**

In the original publication of this article [1], Fig. 3 is wrong, but does not affect discussions and conclusions drawn in the article.

The corrected Fig. 3 is shown below:

**Fig. 3:**
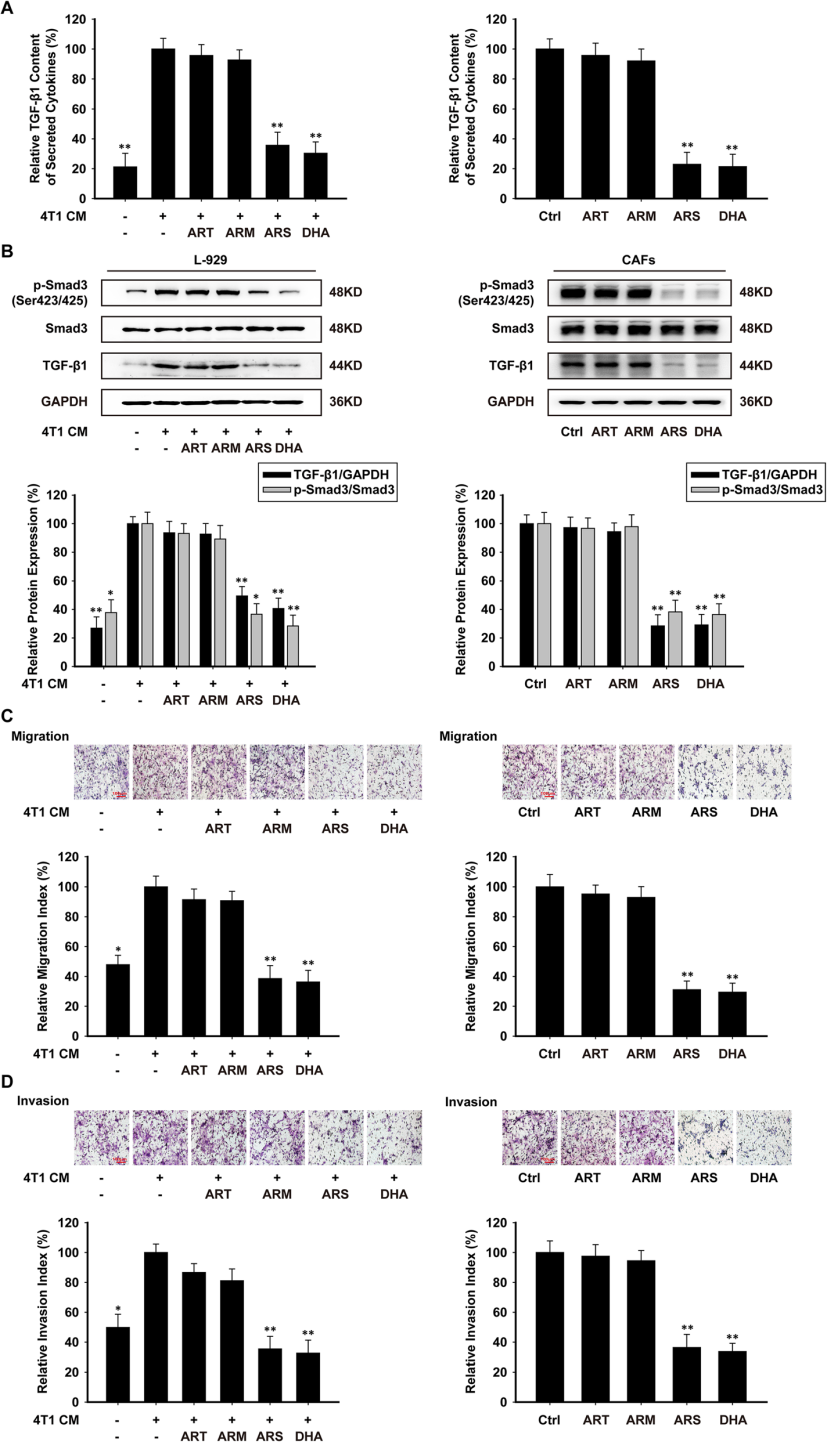
.
